# Rat Islet pECM Hydrogel-Based Microencapsulation: A Protective Niche for Xenotransplantation

**DOI:** 10.3390/gels11070517

**Published:** 2025-07-02

**Authors:** Michal Skitel Moshe, Stasia Krishtul, Anastasia Brandis, Rotem Hayam, Shani Hamias, Mazal Faraj, Tzila Davidov, Inna Kovrigina, Limor Baruch, Marcelle Machluf

**Affiliations:** 1 Faculty of Biotechnology & Food Engineering, Technion—Israel Institute of Technology, Haifa 3200003, Israel; michalskitel@gmail.com (M.S.M.); stasiakrishtul@gmail.com (S.K.); brandis@campus.technion.ac.il (A.B.); shanihm17@gmail.com (S.H.); faratj.mazal@gmail.com (M.F.); tzila937@gmail.com (T.D.); kovrigin@bfe.technion.ac.il (I.K.); baruchl@bfe.technion.ac.il (L.B.); 2 The Interdisciplinary Program for Biotechnology, Technion, Haifa 32000, Israel

**Keywords:** islet microencapsulation, extracellular matrix, insulin delivery, diabetes therapy, biomaterial, artificial pancreas

## Abstract

Type 1 diabetes (T1D) is caused by autoimmune-mediated destruction of pancreatic β-cells, resulting in insulin deficiency. While islet transplantation presents a potential therapeutic approach, its clinical application is impeded by limited donor availability and the risk of immune rejection. This study proposes an innovative islet encapsulation strategy that utilizes decellularized porcine pancreatic extracellular matrix (pECM) as the sole biomaterial to engineer bioactive, immunoprotective microcapsules. Rat islets were encapsulated within pECM-based microcapsules using the electrospray technology and were compared to conventional alginate-based microcapsules in terms of viability, function, and response to hypoxic stress. The pECM microcapsules maintained a spherical morphology, demonstrating mechanical robustness, and preserving essential ECM components (collagen I/IV, laminin, fibronectin). Encapsulated islets exhibited sustained viability and superior insulin secretion over a two-week period compared to alginate controls. The expression of key β-cell transcription factors (PDX1, MAFA) and structural integrity were preserved. Under hypoxic conditions, pECM microcapsules significantly reduced islet apoptosis, improved structural retention, and promoted functional recovery, likely due to antioxidant and ECM-derived cues inherent to the pECM. In vivo transplantation in immunocompetent mice confirmed the biocompatibility of pECM microcapsules, with minimal immune responses, stable insulin/glucagon expression, and no adverse systemic effects. These findings position pECM-based microencapsulation as a promising strategy for creating immunoprotective, bioactive niches for xenogeneic islet transplantation, with the potential to overcome current limitations in cell-based diabetes therapy.

## 1. Introduction

Type 1 diabetes (T1D) is a chronic autoimmune disease where pancreatic β-cells, responsible for secreting insulin in response to high glucose levels, are destroyed by the immune system through T cell-mediated immune response. This chronic autoimmune condition leads to a reduction in β-cell mass, insufficient insulin production, and subsequent dysregulation of blood glucose levels [1,2]. The current treatment of T1D patients is a daily administration of exogenous insulin, coupled with the monitoring of blood glucose levels and a controlled diet [3,4]. Pancreas and pancreatic islet transplantation have been approved, mainly for patients with severe symptoms [5,6,7,8], but this treatment suffers from the shortage of pancreas donors, especially for islet transplantation, where multiple donors are required for a single patient. The donor shortage underscores the potential of xenogeneic islet transplantation as a promising alternative cell source [9,10], though the xenogeneic islets would have to be isolated from the patient’s immune system. One common approach investigated to protect the islets is cell microencapsulation [11]. In this approach, the isolated islets are permanently entrapped in a polymeric network within the confines of a semi-permeable membrane. The membrane selectivity permits the transport of small molecules; thus, nutrients and oxygen can be provided to the immobilized cells, and carbon dioxide and waste products can be removed from the microcapsule. It also enables the secretion of therapeutic factors, such as insulin produced by the islets. On the other hand, this isolated niche restricts the penetration of immunoglobulins and the host immune cells, thus providing immune protection [12,13].

The material used for islet microencapsulation plays a pivotal role in providing the encapsulated cells with biological, structural, and mechanical support, and it generally aims to mimic the natural tissue [14]. It should, therefore, be noncytotoxic to the encapsulated cells and biocompatible with the physiological environment where the microcapsules are to be transplanted. To date, various polymers have been employed for islet encapsulation [15,16], with alginate being the most studied and widely used. Alginate is known for its biocompatibility and ability to provide structural support to encapsulated cells [17]. However, it lacks biofunctional and biomimetic properties that can help regulate cell survival and function [18]. The biomaterial suggested to overcome these obstacles is extracellular matrix (ECM), which presents the natural microenvironment of the cells and exhibits a three-dimensional network of structural proteins, non-structural proteins, and soluble factors [19,20]. The ECM of each tissue is distinguished by a unique composition, organization, and structure [21] that evolves through the intricate interplay between the various resident cells that secrete the ECM molecules [21]. Like other tissues, pancreatic islets are heavily influenced by cell–cell and cell–ECM interactions for their proper function. In mature, intact islets, interactions with the ECM have been shown to regulate multiple aspects of islet physiology, including survival, proliferation, insulin secretion, and preservation of the spherical morphology [22,23,24,25]. Among the predominant ECM components contributing to these regulatory effects are several key proteins. Collagen I, the most abundant fibrous protein in the pancreas, enhances the proliferation, viability, and insulin production of pancreatic β cells [24]. Collagen IV, laminin, and fibronectin positively affect β-cell function, enhancing insulin secretion and cell survival [26,27,28].

Porcine ECM is extensively utilized in biomedical applications due to its broad availability and close structural and biochemical similarity to human ECM, stemming from the evolutionary conservation of its molecular components. Moreover, porcine tissues can be sourced from healthy, well-controlled populations with minimal inter-donor variability. As ECM-based materials gained significant interest in various biomedical applications, decellularization techniques have been developed to remove cellular components and immunogenic epitopes such as α-Gal from tissues, thereby ensuring their safety and functional compatibility while preserving the ECM architecture and composition. Proper pancreas decellularization, thus, can result in a non-immunogenic biocompatible material that preserves the ECM structural, biological, and mechanical properties and may restore substantial cell-ECM interactions [21,29,30].

In the present study, we, therefore, hypothesized that a microencapsulation platform based on pancreatic ECM (pECM) as its sole biomaterial can provide encapsulated rat islets with a pancreatic niche and protect them from the immune system. Applying this approach, we encapsulated rat pancreatic islets in pECM microcapsules and studied the effect of pECM-encapsulation on the encapsulated islets survival and function in vitro and in vivo. Furthermore, we addressed the pECM microencapsulation ability to protect the islets from damage induced under hypoxic conditions and evaluated their biocompatibility following xenotransplantation.

## 2. Results and Discussion

### 2.1. Results

#### 2.1.1. Microcapsules Characterization

**Figure 1:**
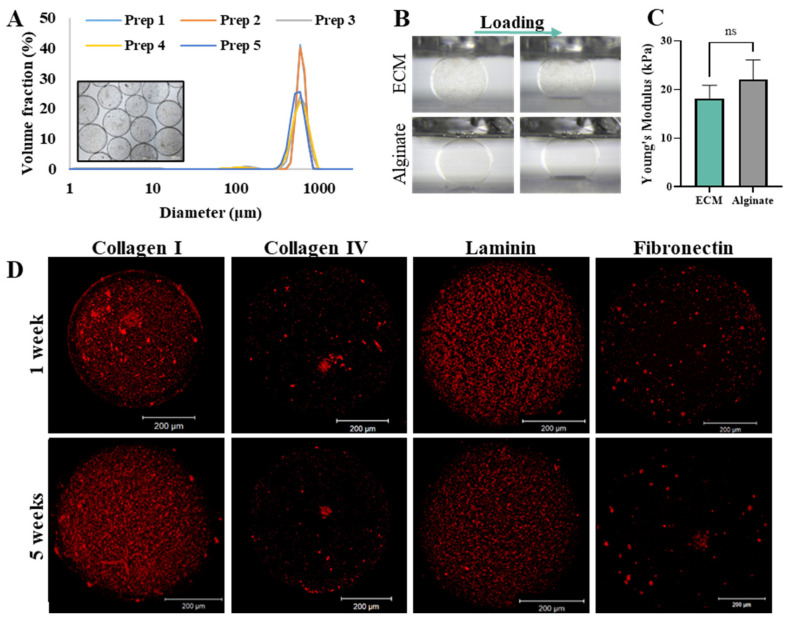
pECM microcapsule characterization. (**A**) Microcapsule size distribution, measured by Malvern Mastersizer 3000 for five independent preparations. (**B**) Mechanical analysis using a microscale tension-compression test system (applied force: 900 µN). (**C**) Calculated Young’s Modulus, based on the tension-compression test (*n* = 6). (**D**) Immunostaining of empty pECM microcapsules for pECM composition: collagen I, collagen IV, laminin, and fibronectin, one- and five-weeks following microcapsules preparation. Scale bars: 200 μm.

pECM microcapsules were fabricated using electrospray technology, exhibiting spherical morphology with a diameter of 619 ± 28 µm and a narrow size distribution that was preserved along multiple preparations (Figure 1A). The mechanical properties of the pECM microcapsules, expressed as Young’s modulus, were not significantly different compared to the widely investigated alginate microcapsules, with values of 18.2 ± 2.6 kPa and 22.1 ± 4.03 kPa, respectively (Figure 1B,C). The presence of the key pECM components following the microcapsules production process and five weeks in culture was addressed through immunostaining. Figure 1D and Supplementary Figure S1 demonstrate that collagen I, known as the predominant protein within pancreatic ECM, alongside collagen IV, laminin, and fibronectin, which play vital roles in the interaction with islets [24], were present in the microcapsules one, two, and 5 weeks following encapsulation.

#### 2.1.2. Encapsulated Islet Characterization

**Figure 2:**
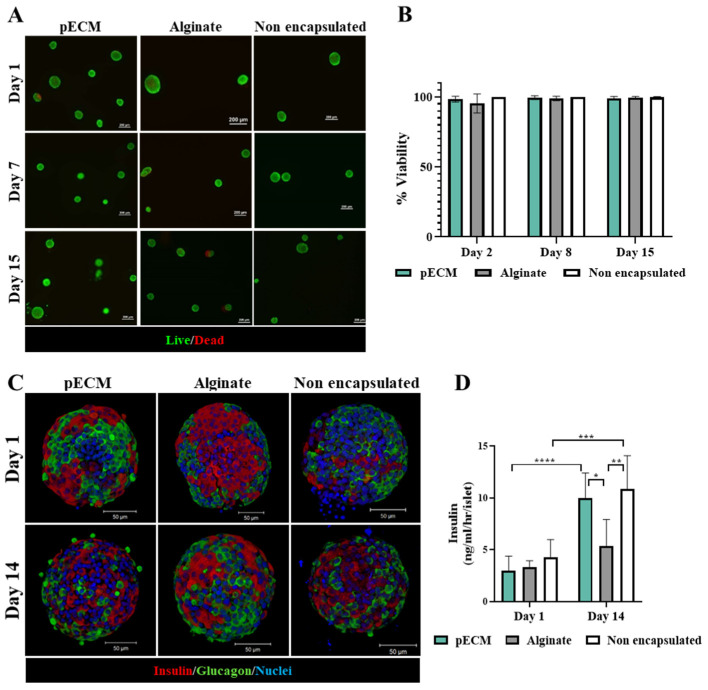
Viability and functionality of pECM-microencapsulated rat islets. (**A**) Fluorescent images of pECM- and alginate-encapsulated islets, and non-encapsulated islets stained with FDA (green, live cells) and PI (red, dead cells) during 15 days of culture. Scale bars: 200 μm. (**B**) Quantified viability results, obtained from DAPI/PI staining images and analyzed using Imaris 9.0.2 analysis software. (**C**) Immunofluorescence staining of insulin (red), glucagon (green), and nuclei (blue) of pECM- and alginate-encapsulated islets, and non-encapsulated islets 1- and 14-days post-encapsulation. Scale bars: 50 µm. (**D**) Insulin secretion upon glucose stimulation (16.7 mM-high glucose) 1- and 14-days post-encapsulation. (* *p* < 0.05, ** *p* < 0.01, *** *p* < 0.001, **** *p* < 0.0001).

After encapsulating rat islets within the pECM microcapsules, we evaluated their viability and functionality during culture, in comparison to alginate-encapsulated islets and non-encapsulated islets. Figure 2A,B demonstrate the high viability of the pECM-encapsulated islets, alginate-encapsulated islets, and non-encapsulated islets with no significant differences between them during the fourteen days of culture. The function of encapsulated islets was addressed through the expression of insulin and glucagon hormones, as well as the glucose-stimulated secretion of insulin. While immunofluorescent analyses demonstrated the expression of both hormones at day 1 and day 14 post-encapsulation by all groups of islets, the insulin secretion analyses revealed notable differences between the function of islets in the different groups (Figure 2C,D and Supplementary Figure S2). On day 1, no significant difference was observed between the groups, followed by a significant improvement in insulin secretion of both pECM-encapsulated and non-encapsulated islets over the 14-day culture period. In the alginate-encapsulated islets, however, no significant increase in the levels of secreted insulin was observed (Figure 2D). Notably, the islets in all groups had a Stimulation index > 1. These results were further supported by a short preliminary in vivo study, which indicated that the pECM-encapsulated islets can secrete insulin following transplantation (Supplementary Figure S3).

**Figure 3:**
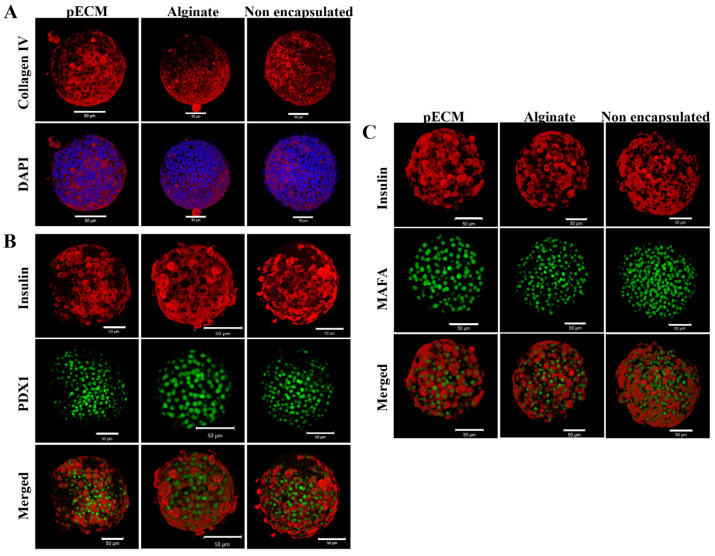
Islet condition and function after fourteen days in culture. Immunofluorescence staining of islets extracted from pECM- and alginate-microcapsules, and non-encapsulated islets 14 days post-encapsulation for (**A**) collagen IV (red) and nuclei (blue), (**B**) insulin (red) and PDX1 (green), and (**C**) insulin (red) and MAFA (green). Scale bars: 50 µm.

Besides evaluating islet viability and functional insulin secretion, we also studied the condition of the encapsulated islets by examining their structural integrity and the expression of functional markers. When addressing the islet structural integrity after 14 days in culture, the expression of collagen IV was observed in all three groups: pECM-encapsulated islets, alginate-encapsulated islets, and non-encapsulated islets (Figure 3A), thus indicating structural integrity preservation [31]. The functional markers that we addressed were PDX1 and MAFA. PDX1 is a transcription factor essential for the maintenance and survival of β-cells [32]. In all three groups, it can be observed that after 14 days in culture, the expression of PDX1 in the cell nuclei is co-localized with beta cells, which’s cytoplasms are stained for insulin, thus confirming that beta cells maintain the expression of PDX1 (Figure 3B). MAFA is another transcription factor necessary for adult β-cell function and the loss of its expression is considered the first indication of islet deterioration [33]. Figure 3C shows that after 14 days of culture, MAFA expression persists in the β-cells of the islets from all three groups.

#### 2.1.3. pECM Microcapsules Protect the Islets from Hypoxia Damage

**Figure 4:**
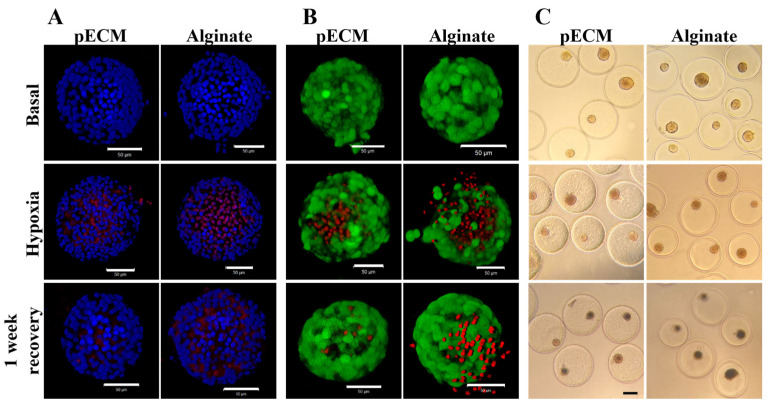
The recovery of encapsulated rat islets following hypoxia. Confocal fluorescent microscopy images of pECM- and alginate-encapsulated islets, stained with (**A**) DAPI (nuclei, blue), PI (apoptotic cells, red) and (**B**) FDA (viable cells, green), PI (apoptotic cells, red) at different time points: basal, after 24 h hypoxia, and following 1 week of recovery. (**C**) Bright-field images of pECM- and alginate-encapsulated islets, taken at basal condition, after 24 h hypoxia, and following 1 week of recovery. Scale bars: 200 μm.

Hypoxia stress represents a significant challenge that islets face post-transplantation, especially for beta cells, which are highly sensitive to oxygen deprivation [11,22]. To investigate the potential of the bioactive pECM microcapsules in reducing stress-induced damage to the encapsulated islets, we investigated the islets under hypoxic conditions. In these studies, pECM- and alginate-encapsulated islets, and non-encapsulated islets were subjected to hypoxia for 24 h, followed by a week-long recovery period, and assessed for their viability and functionality. The non-encapsulated islets did not survive the hypoxic conditions, resulting in complete dissociation of the islets that precluded any further analysis. The survival of the pECM- and alginate-encapsulated islets was evaluated at three time points: basal, immediately after hypoxia, and after 1 week of recovery. Apoptotic cells were stained and compared to the entire cell population or to living cells. Representative confocal images provided a qualitative visualization of the damage induced by hypoxia, revealing more severe damage to the alginate-encapsulated islets compared to the pECM-encapsulated islets (Figure 4A,B). Moreover, after 1 week of recovery, the pECM-encapsulated islets exhibited a higher recovery rate compared to those encapsulated in alginate. Another phenomenon that could be observed in these experiments was the loss of islets’ structural integrity, a crucial factor for their proper function. While the pECM-encapsulated islets preserved their structural integrity throughout the experiment, in the alginate-encapsulated islets, we observed a dissociation of cells from the islets following the induction of hypoxic conditions. Bright-field images enabled the discernment of differences between the groups after 1 week of recovery. While in the alginate group, a predominant observation was the presence of black necrotic cores in most of the islets, in the pECM-encapsulated islets, this phenomenon was both less frequent and less severe (Figure 4C).

**Figure 5:**
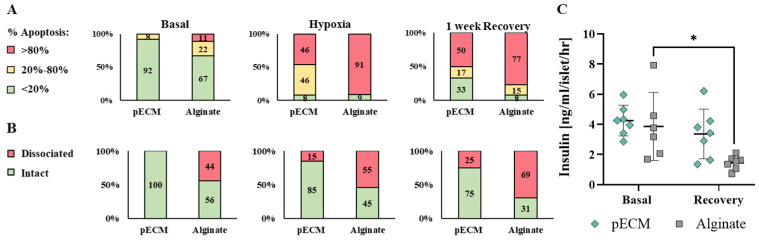
Quantified hypoxia-induced damage and recovery of encapsulated rat islets. (**A**) Percentage of pECM- and alginate-encapsulated islets, classified according to their apoptotic ratio: severe damage of >80% apoptotic cells (red), moderate damage of 20–80% apoptotic cells (yellow), and good condition of <20% apoptotic cells (green), at basal time point, after 24 h of hypoxia, and following 1 week of recovery. (**B**) Percentage of intact (green) and dissociated islets (red), before hypoxia (basal), after 24 h of hypoxia, and following 1 week of recovery. (**C**) Insulin secretion upon glucose stimulation (16.7 mM-high glucose) under basal conditions and after 1 week of recovery from 24 h of hypoxic condition, comparing pECM- and alginate-encapsulated islets. (* *p* < 0.05).

To quantify the damage caused by the hypoxic conditions, we classified the islets according to their apoptotic ratio. The islets were divided into three groups: (1) islets in poor condition with over 80 percent apoptotic cells (presented in red), (2) moderately damaged islets with apoptotic cell percentages ranging from 20 to 80 percent (presented in yellow), and (3) islets in good condition with less than 20 percent apoptotic cells (presented in green). Figure 4 reveals that some apoptotic islets were already evident under basal conditions in the alginate group. Upon induction of hypoxia, the severe damage to the islets resulted in over 90 percent of highly apoptotic islets in the alginate group and nearly 50 percent in the pECM-encapsulated islets. Following 1 week of recovery from hypoxia, however, partial recovery was observed in the alginate-encapsulated islets, with fewer islets in poor condition (77 percent) and 15 percent of moderately damaged islets. A higher recovery rate was observed in the pECM-encapsulated islets, with 33 percent of islets in good condition compared to 8 percent immediately after hypoxia (Figure 5A). To quantify the islet structural integrity following hypoxia, the islets were classified into intact or dissociated islets. Figure 5B demonstrates that at the basal time point, the alginate group already had more than 40 percent dissociated islets, compared to the pECM group, where no islet dissociation was observed. Following the induction of hypoxia, dissociation was also seen in 15 percent of the pECM group, while in the alginate group, the percentage of dissociated islets increased to 55 percent. Following one week, the percentage of dissociated islets further increased to 25 percent in the pECM-encapsulated islets and almost 70 percent in the alginate-encapsulated islets (Figure 5B). Another measure of hypoxic damage and islet recovery following hypoxia is islet functionality in terms of glucose-responsive insulin secretion. After one week of recovery, the alginate-encapsulated islets demonstrated a significant decrease in insulin secretion compared to the basal condition. In contrast, the pECM-encapsulated islets had recovered, thus secreting insulin levels that were not significantly different from the basal condition (Figure 5C and Supplementary Figure S4).

#### 2.1.4. Biocompatibility of pECM Microcapsules

**Figure 6:**
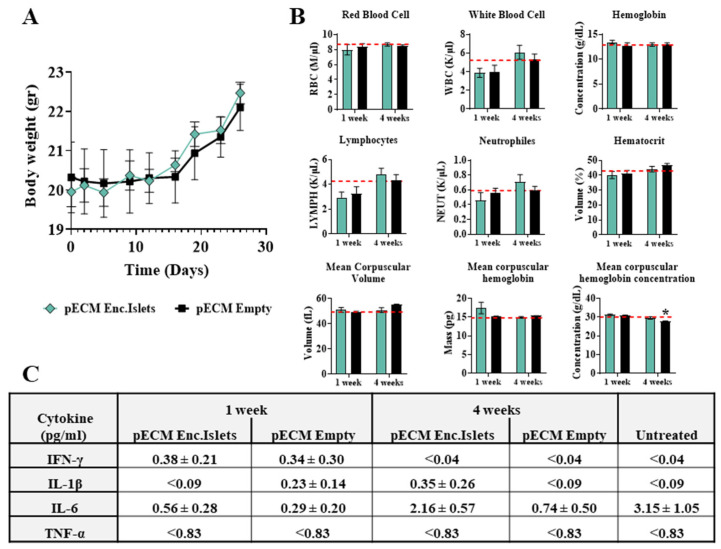
In vivo biocompatibility studies. pECM-encapsulated islets and empty pECM microcapsules were transplanted into immunocompetent mice, and the inflammatory reactions were followed. (**A**) Mice’s body weight measurements throughout the experiment. (**B**) Complete blood count at 1 and 4 weeks following microcapsule transplantation. Red dashed lines represent the mean of normal blood values for healthy, untreated mice (* *p* < 0.05 to the untreated group). (**C**) Cytokine levels in the serum 1 and 4 weeks post-transplantation compared to untreated mice. The values are presented as mean ± SEM.

The biocompatibility of pECM-encapsulated rat islets was evaluated in vivo through their subcutaneous transplantation to immunocompetent mice in comparison to empty pECM microcapsules. The mice were followed for their body weight during the experiment, showing a normal increase with no significant difference between the groups (Figure 6A). One and four weeks post-transplantation, the mice were sacrificed, and their blood was taken for a complete blood count (CBC) analysis. The results were compared to normal values of untreated healthy mice and showed similar profiles between the groups, with no significant differences between the groups, which indicated no significant inflammatory reaction (Figure 6B). Furthermore, the inflammatory cytokines INF-γ, IL1-β, IL-6, and TNF-α levels in the serum exhibited comparable results with no significant differences between the groups and compared to untreated mice, 1 and 4 weeks following transplantation (Figure 6C).

**Figure 7:**
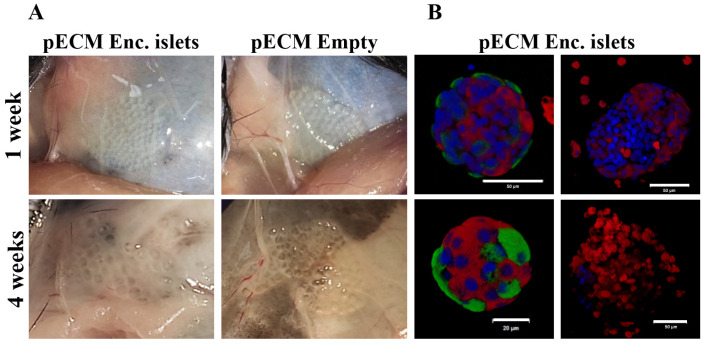
Biocompatibility of the implanted microcapsules. (**A**) pECM-encapsulated islets and empty pECM microcapsules at the transplantation site, 1 and 4 weeks following transplantation. (**B**) pECM-encapsulated islets 1 week and 4 weeks following transplantation, immunostained for insulin (red) and glucagon (green). Nuclei are stained using DAPI (blue). Scale bars: 50 µm.

When examining the microcapsules at the implantation site 1 and 4 weeks after transplantation, a clear visualization of their spherical morphology is seen, with no significant difference between the groups in terms of the degree of tissue overgrowth (Figure 7A and Supplementary Figure S5). Examination of the encapsulated islet function by assessing the expression of the hormones insulin and glucagon at these two time points indicates heterogeneity, with some islets expressing both hormones, while others express only insulin (Figure 7B).

### 2.2. Discussion

The development of bioartificial pancreas represents a promising frontier in diabetes therapy, offering a potential solution to the challenges associated with conventional insulin therapy and pancreas transplantation [34]. One of the important factors influencing the success of a bioartificial pancreas is the selection of bioactive materials, which play a crucial role in creating a supportive microenvironment that promotes the survival, function, and long-term efficacy of the encapsulated cells. Therefore, ECM-based materials can potentially offer a biomimetic microenvironment that closely mimics the native tissue, facilitating vital interactions with encapsulated cells [21]. Given the variation in ECM composition across different tissues, each tailored to their specific physiological functions, our focus has been on utilizing porcine pancreatic ECM for islet microencapsulation. This approach aims to provide the encapsulated islets with a natural pancreatic microenvironment.

Following the optimization steps, we successfully generated spherical microcapsules, exhibiting a nonsignificant difference in Young’s modulus from the widely used alginate microcapsules. Achieving a spherical morphology of the microcapsules while ensuring their mechanical stability is a pivotal determinant affecting both the viability of encapsulated cells and the success of the transplantation procedure [35]. Hence, irregular shapes or broken microcapsules are associated with undesirable cell penetration and inflammatory response [11,35]. The microcapsules should also demonstrate adequate mechanical resistance to withstand the shear forces encountered during the implantation process, as well as the internal pressure that the enclosed cells may generate [35]. Additionally, each potential transplantation site presents exposure to varying enzymes, pH fluctuations, inflammatory response, mechanical disturbance, and other forces exerted by the surrounding tissues, significantly impacting the stability and function of transplanted microcapsules [36,37,38,39]. A major contribution to microcapsules’ mechanical resistance is attributed to poly-L-lysine (PLL) coating, which is present in both pECM and alginate microcapsules [40,41]. Indeed, Young’s modulus of both alginate and pECM microcapsules was in the range of 10–50 kPa, thus suggesting they offer adequate structural support for the encapsulated islets, endure the pressure generated during injection, and maintain stability in the transplantation site over an extended period, as was previously reported and further shown in our in vivo biocompatibility studies [40,41,42].

Besides the mechanical support to the encapsulated islets, the pECM microcapsules can offer a significant advantage in terms of biological functionality. This biological support is facilitated by proteins naturally found in the pancreas, such as collagen I, collagen IV, fibronectin, and laminin, which were demonstrated to remain in the microcapsules for at least five weeks in culture. Collagen I, the most abundant fibrous protein in the pancreas, enhances the proliferation, viability, and insulin production of pancreatic β cells [24]. Collagen IV, laminin, and fibronectin positively affect β-cell function, enhancing insulin secretion and cell survival [26,27,28]. The presence of these proteins in the pECM microcapsules may have, thus, contributed to the high viability presented by islets encapsulated within the pECM microcapsules, as well as to their significant improvement in insulin secretion over two weeks. We have also demonstrated the maintenance of islet structural integrity and β-cell functionality through the detection of collagen IV within the intra-islet ECM, and the two essential transcription factors, PDX1 and MAFA, following a 14-day culture period, in pECM-encapsulated islets as well as in alginate-encapsulated islets and non-encapsulated islets. Collagen IV forms a three-dimensional structure that interacts with other crucial basement membrane components, important in maintaining proper basement membrane morphology and microstructural integrity [31,43,44]. The transcription factors PDX1 and MAFA play crucial roles in regulating the transcription of genes responsible for glucose sensing, insulin secretion, and the maintenance of proper function in adult β-cells [32,33,45]. Deterioration in β-cell function is correlated with an earlier decrease of MAFA expression and its DNA binding activity compared to Pdx1 [46]. The effect on PDX1 activity is likely influenced by alterations in nuclear localization [47], whereas the inhibition of MAFA involves processes such as mRNA processing, stability, and cellular localization [47,48]. Hence, the presence of these transcription factors in β-cell nuclei of the encapsulated and non-encapsulated islets signifies the proper functioning of β-cells.

Previous research has similarly demonstrated a significant improvement in encapsulated islet function with the incorporation of ECM components, such as collagen IV, laminin, fibronectin, and RGD binding motif, within the microcapsules [18,49,50]. Interestingly, researchers showed that the balance between the ECM components has a crucial influence on the islet function [26,51,52]. For example, a high concentration of collagen type IV, above the physiological concentration, was reported to reduce insulin secretion of encapsulated islets [26]. Furthermore, it is well established that during the islet isolation procedure, the islet-ECM interactions are damaged and are not fully restored following encapsulation and transplantation [53,54]. Therefore, our platform aims to offer enhanced support for islet function and recovery by providing the physiological ECM composition, which is likely damaged during isolation.

The beneficial effect of our pECM microcapsules was also manifested by protecting the encapsulated islets under the stress of hypoxia. Upon transplantation, pancreatic islets encounter various challenges, including hypoxic stress, which can significantly impact their survival and functionality. This stress emerges from insufficient oxygen supply to the transplanted islets, particularly impacting the β-cells due to their high sensitivity to oxygen deficiency. Consequently, cellular dysfunction, impaired insulin secretion, and eventual graft failure may occur [11,50]. The pECM-encapsulated islets demonstrated a reduced number of apoptotic cells immediately following hypoxia induction, with a higher recovery rate than alginate-encapsulated islets one week after hypoxia. Furthermore, a greater preservation of islet structural integrity and recovery of functional insulin secretion was observed in the pECM group.

These findings are supported by several studies investigating the effects of ECM component incorporation into scaffolds, as well as the provision of antioxidant proteins and molecules to islets, on their survival under hypoxic conditions [50,55,56,57,58]. Zbinden et al. Demonstrated that the damage induced by hypoxia on the expression of basement membrane proteins and overall β-cell functionality was significantly reduced with the addition of protective collagen I hydrogel, underscoring the importance of biological and physical support provided by collagen I [55]. Similarly, Brandhorst et al. showed the protective impact of single basement membrane proteins on islet function under hypoxic conditions, which was enhanced with the combination of multiple proteins and concentration-dependent [50]. Most importantly, an even greater protective effect was obtained when providing the islets with soluble ECM [22].

We have previously shown the protective effect of metallothionein (MT1E, MT1F) and Scavenger Receptor Class A Member 3 (SCARA3)–preserved in the pECM during the decellularization process–under proinflammatory cytokine exposure [59]. They function by scavenging various reactive oxygen species (ROS) such as superoxide, hydrogen peroxide, hydroxyl radical, and nitric oxide release following hypoxia and oxidative stress [60,61]. Their presence within the pECM microcapsules likely contributed to the enhanced performance of pECM-encapsulated islets under hypoxic stress, compared to those encapsulated in alginate.

The aspect of microcapsules’ biocompatibility is predominant for ensuring graft survival, since activation of the innate immune system can lead to the release of harmful cytokines detrimental to the encapsulated cells [62]. This biocompatibility rests on the microcapsules’ polymeric composition and the encapsulated cellular component. Previous research in our lab has already validated the pECM’s biocompatibility, by comparing empty pECM microcapsules with the most investigated alginate microcapsules, showing no elicitation of an immune response [63]. However, the cellular component, referring to the encapsulated cell type, can significantly influence the immune response due to the release of proteins and metabolic products from the microcapsules, alongside cellular components cleared from dead cells into the transplantation site [64,65,66]. This aspect is particularly pertinent in the context of xenotransplantation [64,67]. In our studies, the biocompatibility of pECM-encapsulated islets was manifested in normal weight gain, similar CBC profiles compared to the empty pECM microcapsules control, and comparable serum cytokine levels. Most importantly, the transplanted islets’ capability to produce insulin was retained, thereby indicating that no severe functional damage was caused.

## 3. Conclusions

This study highlights the significant advantages of the developed pECM microencapsulation platform, which closely mimics the physiological conditions of native pancreatic tissue and provides a complex, supportive microenvironment for encapsulated islets. The pECM microcapsules preserved islet viability and structural integrity, supported superior insulin secretion, and promoted recovery following hypoxic stress. The in vivo studies further demonstrated their biocompatibility, confirming the potential of this approach as an effective and sustainable strategy for xenogeneic islet transplantation. These findings position pECM-based microencapsulation as a promising solution for overcoming current limitations in islet transplantation and advancing cell-based therapies for diabetes.

## 4. Materials and Methods

All animal experiments were performed in compliance with the Council of Animal Experiments and the Israel Ministry of Health guidelines for the care and use of laboratory animals and approved by the Animal Ethics Committee at the Technion, Israel. The number of animals per group was determined based on a power analysis with 1 − β = 0.8, α = 0.5. Animals were purchased from Envigo (Jerusalem, Israel).

### 4.1. Rat Islet Isolation

Islets were isolated from Sprague Dawley (SD) rats weighing 200–240 gr. The rats were euthanized using CO_2_, and an abdominal midline incision was made. The bile duct was cannulated with a 22-gauge catheter, and collagenase P solution (1 mg/mL, Roche, Mannheim, Germany) was injected into the pancreas. The harvested pancreas was digested at 37 °C, and the islets were separated on a discontinuous Histopaque density gradient (1.119 g/mL and 1.077 g/mL, Sigma, Rehovot, Israel). The islets were further purified by handpicking under a binocular and cultured in RPMI-1640 medium supplemented with 25 mM HEPES (Sigma, Rehovot, Israel), 2 mM GlutamaX, 10% FBS (Gibco, New York, NY, USA), and 1% Pen-Strep^®^ (Biological Industries, Beit-Haemek, Israel), under 5% CO_2_ and 37 °C.

### 4.2. Preparation of pECM Biomaterial

The pancreatic ECM decellularization and solubilization were performed according to a protocol we previously established [59,63,68]. Briefly, pancreases of healthy commercial juvenile pigs (~6 months old, Lahav C.R.O, Negev, Israel) were sequentially treated with alternating hypertonic/hypotonic solutions, trypsin–EDTA (Sigma-Aldrich, Rehovot, Israel), and Triton X-100 (Merck Millipore, Burlington, MA, USA). The decellularized pancreases were then lyophilized, cut into pieces of about 1 × 1 mm, and enzymatically solubilized by pepsin (Sigma-Aldrich, Rehovot, Israel).

### 4.3. Microencapsulation of Islets

Isolated rat islets were suspended in serum-free growth media and mixed with 4% (*w*/*v*) sodium alginate (PRONOVA UP MVG, NovaMatrix, Sandvika, Norway) and 2.1% (*w*/*v*) solubilized pECM, resulting in final concentrations of 1.6% (*w*/*v*) and 0.26% (*w*/*v*), respectively. For alginate microcapsules, the suspended islets were mixed with alginate 4% (*w*/*v*) to achieve a final concentration of 1.6% (*w*/*v*). Microspheres were produced using electrospray technology. The islet suspension was injected through a 25-gauge needle at a constant flow rate of 0.198 mL/min (syringe pump, Harvard Apparatus, Holliston, MA, USA) and a voltage of 6.5 kV, into a 130 mM CaCl_2_ solution, where it was allowed to gel for 20 min. Subsequently, the microcapsules were coated with 0.06% poly-L-lysine (PLL, Sigma, Rehovot, Israel) and incubated for 30 min at 37 °C to allow pECM crosslinking. Then, the alginate in the microcapsules’ core was liquified using 1.5% (*w*/*v*) sodium citrate solution, and the encapsulated islets were cultured in RPMI-1640 medium, supplemented with HEPES, 2 mM GlutamaX, 10% FBS, and 1% Pen-Strep^®^.

### 4.4. Microcapsules Characterization

#### 4.4.1. Size Distribution Analysis

Microcapsule size distribution was measured using a diffraction particle size analyzer, Mastersizer 3000 (Malvern Instruments Ltd., Worcestershire, UK). The diameter mean was calculated based on D[4,3]—volume-weighted mean, based on five independent preparations.

#### 4.4.2. Mechanical Properties

The mechanical properties of the microcapsules were analyzed using a microscale mechanical test system (MicroTester MT G2, CellScale, Waterloo, ON, Canada). A force of 900 µN was applied through a 0.3 mm diameter beam attached to a 2 × 2 mm compression plate. The microcapsules underwent a loading duration of 60 s, followed by a 30-second recovery period, and the same cycle was repeated. The compression force and displacement were recorded continuously, and the Young’s modulus was calculated based on the recorded data. The results were derived from at least four independent samples.

#### 4.4.3. Immunostaining Microcapsules and Encapsulated Islets

For the immunostaining of microcapsules’ components, microcapsules were fixed in paraformaldehyde (PFA, 4%) overnight, followed by permeabilization with 1% Triton X-100 for 1 h, and blocking of unspecific protein binding sites through 1 h incubation in 10% fetal bovine serum and 0.1% Triton X-100 in PBS. The microcapsules were immunostained with antibodies for collagen type I (1:100, Sigma-Aldrich #C2456), collagen type IV (1:100, Abcam, Cambridge, UK #ab6586), laminin (1:100, Sigma-Aldrich #L8271), and fibronectin (1:100, Santa Cruz Biotechnology #sc-81767). For the immunostaining of encapsulated islets, the fixed islets were extracted from the microcapsules, permeabilized using 1% Triton X-100 for 20 min, and then incubated for 1 h in 10% fetal bovine serum and 0.1% Triton X-100 in PBS to block unspecific protein binding sites. The islets were immunostained with antibodies against insulin (1:50, Abcam #ab7842, or 1:200, Abcam, # ab181547), glucagon (1:500, Abcam #ab10988), collagen type IV (1:100, Abcam #ab6586), PDX1 (1:100, cell signaling, #5679), and MAFA (1:1000, cell signaling, #79737). DAPI (0.5 µg/mL, Biotium #40011) was used for DNA staining. Images were taken using LSM700 and LSM710 confocal microscopes (Carl Zeiss, Jena, Germany).

### 4.5. Islet Viability

Islet viability was qualitatively assessed through staining with fluorescein diacetate (FDA, 10 μg/mL, Sigma, Rehovot, Israel) and propidium iodide (PI, 10 μg/mL, Sigma, Rehovot, Israel) for 10–15 min in the dark. Samples were imaged using a fluorescent microscope (Nikon TE2000-E, Nikon, Japan) or a confocal microscope (LSM700 and LSM710, Carl Zeiss, Jena, Germany).

For viability quantification, islets were stained with PI (10 μg/mL, Sigma, Rehovot, Israel) for 10–15 min in the dark, followed by fixation in 4% PFA for 30 min and nuclei staining with DAPI (0.5 µg/mL, Biotium, Fremont, CA, USA) for 20 min. The stained islets were imaged using a confocal microscope (LSM700 and LSM710, Carl Zeiss, Oberkochen, Germany). Viability was quantified using Imaris software and calculated as the percentage of cells stained with DAPI but not with PI.

### 4.6. Glucose-Stimulated Insulin Secretion Analysis

Islets were incubated in Krebs–Ringer Buffer (KRB) for 1 h (*n* = 6). Then, islets were transferred to a low glucose solution (2.8 mM glucose in KRB solution) for 1 h, followed by incubation in a high glucose solution (16.7 mM glucose in KRB solution) for another 1 hr. Media was sampled after incubation in the low glucose solution and the high glucose solution. The levels of insulin secreted to the media were quantified using a rat ultrasensitive insulin ELISA kit (Alpco, Salem, MA, USA) according to the manufacturer’s instructions and expressed as ng/mL/islet/hr. Stimulation index: ratio between insulin secreted under 16.7 mM glucose to insulin secreted under 2.8 mM glucose.

### 4.7. Effect of Hypoxia on Islet Survival and Function

To induce hypoxia, pECM- and alginate-encapsulated islets were incubated under 2% O_2_ within a modular incubator chamber (MIC-101, Billups-Rothenberg Inc., San Diego, CA, USA). The chamber was flushed with a gas mixture comprising 93% N2, 5% CO_2_, and 2% O_2_ for several minutes, and then sealed and placed in a 37 °C incubator for 24 h. Subsequently, 20 islets were sampled and stained with FDA-PI, and another 20 islets were stained with DAPI-PI for viability assessment, as detailed above. The media was replaced for the remaining islets, and after a recovery period of 1 week, viability and functional insulin secretion were evaluated as detailed above. The results are derived from three independent experiments, with one experiment selected as representative.

### 4.8. Biocompatibility pECM-Encapsulated Islets

Eight-week-old C57B6/J male mice were subcutaneously injected with either pECM-encapsulated rat islets or with empty pECM microcapsules as a control (*n* = 6 mice per group, randomly allocated). Untreated mice were utilized to determine the basal level of the examined parameters. The mice were weighed twice a week and were sacrificed one and four weeks following microcapsule injection. At each scarification time point, mice were weighed, blood was taken, and the microcapsules were imaged in the injection site and retrieved for further analyses. Blood was taken for a complete blood count (CBC) analysis performed by AML Laboratories, Israel. Cytokines in the serum (IL-1β, IL-6, INF-γ, and TNF-α) were measured using Milliplex Mouse Cytokine/Chemokine Magnetic Bead Panel (MCYTOMAG-70K-03, Millipore) according to the manufacturer’s protocol. The microcapsules were retrieved to evaluate their morphology and tissue overgrowth, as well as insulin and glucagon expression by the encapsulated islets through immunostaining and H&E staining histological analyses. Confounders were not controlled, and the group allocation was known at all stages. Data points that deviated by more than 20% from the mean were excluded from the analysis, but only if this resulted in the removal of less than 10% of the data points.

### 4.9. Statistical Analysis

All statistical analyses were conducted using GraphPad Prism v.10 Software. Results are expressed as mean ± standard deviation (SD) for at least three repetitions per experimental group and time point. For the in vivo biocompatibility studies, data are presented as mean ± standard error of the mean (SEM). Statistical differences between means were assessed using a t-test for individual comparisons or two-way ANOVA, followed by Tukey’s multiple comparisons test. A *p*-value of less than 0.05 was considered statistically significant.

## Data Availability

The original contributions presented in this study are included in the article/Supplementary Materials. Further inquiries can be directed to the corresponding author.

## Supplementary Materials

The following supporting information can be downloaded at https://www.mdpi.com/article/10.3390/gels11070517/s1, Figure S1: pECM microencapsulated islet immunostaining for collagen I (green) and nuclei (blue) fourteen days post-encapsulation. Scale bar: 200 μm; Figure S2: Insulin secretion upon glucose stimulation (low glucose: 2.8 mM, high glucose: 16.7 mM) one- and fourteen-days post-encapsulation. (* *p* < 0.05, ** *p* < 0.01, *** *p* < 0.001, **** *p* < 0.0001); Figure S3: Mice blood glucose following xenotransplantation of pECM-encapsulated rat islets, alginate-encapsulated islets, or empty pECM microcapsules; Figure S4: Insulin secretion upon glucose stimulation (low glucose: 2.8 mM, high glucose: 16.7 mM) under basal conditions and after 1 week of recovery from 24 h of hypoxic condition, comparing pECM- and alginate-encapsulated islets. (* *p* < 0.05); Figure S5: H & E staining of pECM-encapsulated islets (Enc. islets) and empty pECM microcapsules (Empty) 1 week after transplantation. (Scale bar: 500 μm).
