# Supplementary material for: Rat Islet pECM Hydrogel-Based Microencapsulation: A Protective Niche for Xenotransplantation
Source: Gels. 2025 Jul 2;11(7):517. doi: 10.3390/gels11070517 (PMC12295616; doi:10.3390/gels11070517)
Supplement: Supplementary file 1 [file gels-11-00517-s001.zip › gels-3700644-supplementary.pdf]

gels-3700644

Rat Islet pECM Hydrogel-Based Microencapsulation: A Protective Niche for Xenotransplantation

*Michal Skitel Moshe, Stasia Krishtul, Anastasia Brandis, Rotem Hayam, Shani Hamias, Mazal Faraj, Tzila Davidov, Inna Kovrigina, Limor Baruch, and Marcelle Machluf**

Supplementary Data

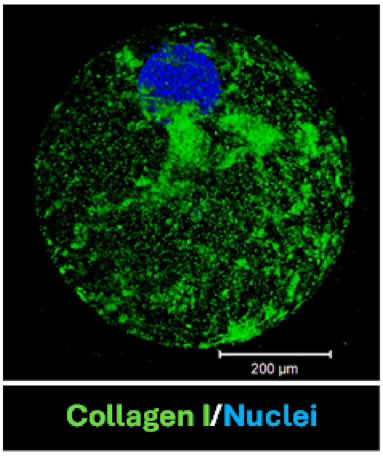

Supplementary Figure S1: pECM microencapsulated islet immunostaining for collagen I (green) and nuclei (blue) fourteen days post-encapsulation. Scale bar: 200µm.

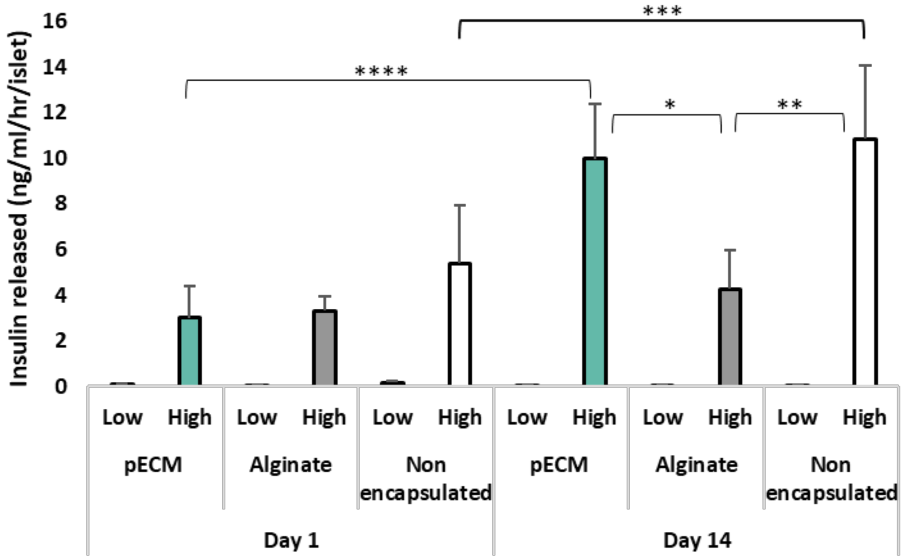

Supplementary Figure S2: Insulin secretion upon glucose stimulation (Low glucose: 2.8mM, High glucose: 16.7 mM) one- and fourteen-days post-encapsulation. (*$p<0.05$, **$p<0.01$, ***$p<0.001$, ****$p<0.0001$).

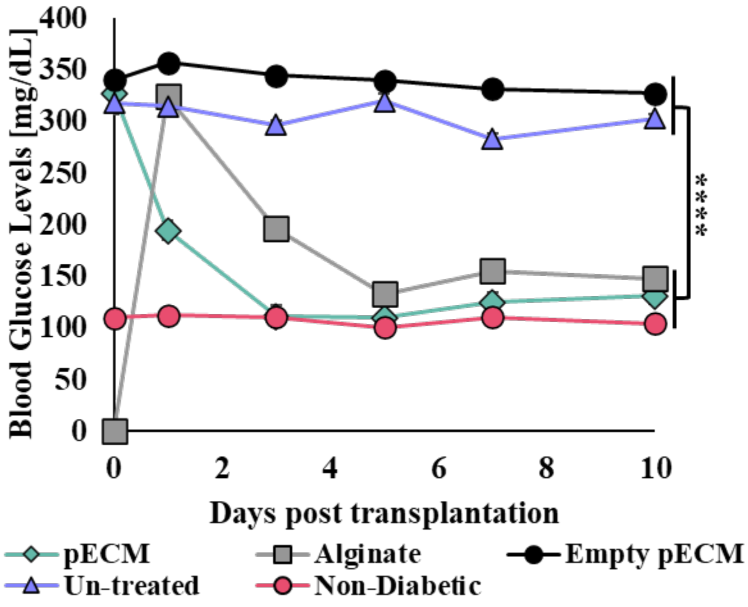

Supplementary Figure S3: Mice blood glucose following xenotransplantation of pECM-encapsulated rat islets, alginate-encapsulated islets, or empty pECM microcapsules.

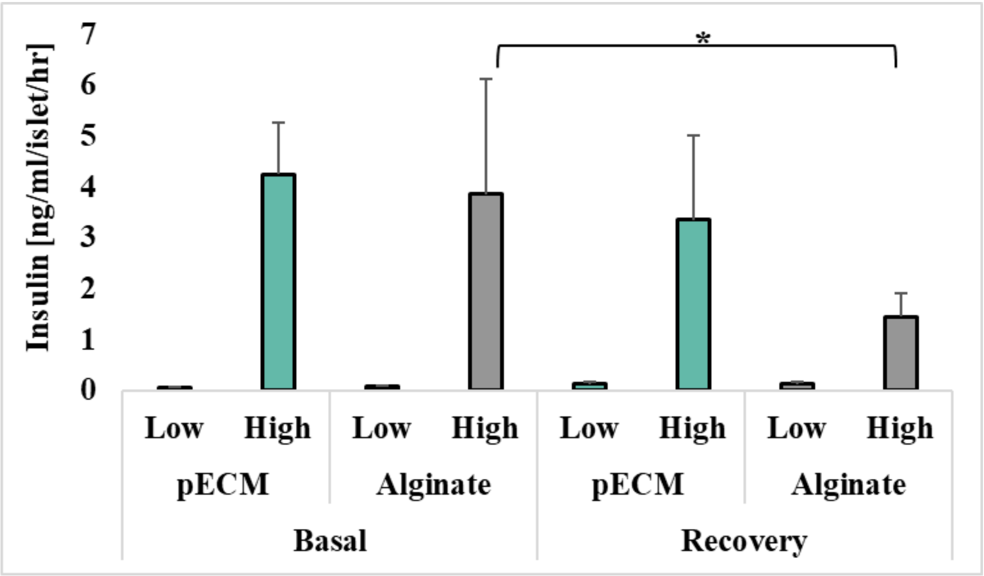

Supplementary Figure S4: Insulin secretion upon glucose stimulation (Low glucose: 2.8mM, High glucose: 16.7 mM) under basal conditions and after 1 week of recovery from 24 hours of hypoxic condition, comparing pECM- and algi-nate-encapsulated islets. (*p<0.05).

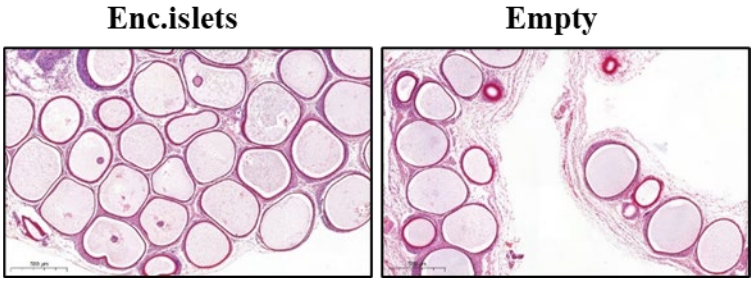

Supplementary Figure S5: H&E staining of pECM-encapsulated islets (Enc.islets) and empty pECM microcapsules (Empty) 1 week after transplantation. (Scale bar: 500µm).

Supplementary Methods

In vivo efficacy evaluation of pECM-encapsulated islets: To induce diabetes, C57B6/J male mice (18-24 gr) were intraperitoneally (IP) injected with streptozotocin (STZ, 60 mg/kg body weight), dissolved in ice-cold 0.1M citrate buffer pH 4.5, for 5 consecutive days. Mice's blood glucose levels were measured three times a week at a constant time during the day without fasting, using a FreeStyle glucometer (Abbott Diabetes Care). Mice were considered to have diabetes when their blood glucose levels were consistently measured with 300 mg/dL or higher for a minimum of two consecutive measurements. Diabetic mice were randomly divided into treatment groups and were subcutaneously injected with the microcapsules. The treatment groups were as follows: pECM-encapsulated rat islets, alginate- encapsulated rat islets, and empty pECM microcapsules. Untreated diabetic mice and healthy mice were used as negative and positive controls, respectively. During the experiment, the mice were followed for their weight, blood glucose levels, and vitality three times a week.
